# Upcycling Orange-Derived Peel and Bagasse into ZnCl_2_‑Activated Biochar for Sustainable Enrofloxacin Removal

**DOI:** 10.1021/acsomega.6c00992

**Published:** 2026-06-26

**Authors:** Kleryton Luiz Alves de Oliveira, Ana Luíza Ferreira de Matos, Marcela de Oliveira Brahim Cortez, Antonio Machado Netto, Noemí Cristina Silva de Souza, Angélica de Cassia Oliveira Carneiro, Renata Pereira Lopes Moreira

**Affiliations:** † Department of Chemistry, 28120 Universidade Federal de Viçosa, Peter Henry Rolfs Avenue, s/n, University Campus, Viçosa 36570-900, MG, Brazil; ‡ Department of Forest Engineering, 28120 Universidade Federal de Viçosa, Peter Henry Rolfs Avenue, s/n, University Campus, Viçosa 36570-900, MG, Brazil

## Abstract

The sustainable management of agro-industrial residues, aligned with the United Nations Sustainable Development Goals (SDGs), has emerged as an effective strategy for mitigating environmental impacts and enabling low-cost environmental technologies. In this context, a ZnCl_2_-activated biochar was synthesized from orange peel and orange bagasse agro-industrial residues and evaluated for the removal of emerging contaminants from aqueous systems, using enrofloxacin as a model compound. The activated biochar exhibited a high fixed carbon content (63.1 wt %) and ash content of 14.74 wt %, along with a decrease in the H/C and O/C atomic ratios compared to the raw biomass, indicating increased carbonization and aromaticity. FTIR analysis showed the loss of oxygenated (O–H and C–O) and aliphatic groups after pyrolysis, along with the emergence of aromatic C=C structures, indicating increased aromaticity. The biochar showed a remarkable increase in specific surface area, reaching 1140 m^2^ g^–1^, compared to 0.475 m^2^ g^–1^ for the precursor biomass. SEM analyses revealed a rough, fragmented, cavity-rich surface morphology. XRD confirmed the predominantly amorphous structure of the biochar. Adsorption assays demonstrated nearly complete enrofloxacin removal, with kinetics well described by the Elovich model and equilibrium data fitting the Freundlich isotherm. The biochar also maintained satisfactory performance in the presence of interfering species commonly found in lagoon water. Overall, these results highlight the strong potential of citrus-waste-derived activated biochar as a sustainable adsorbent for emerging contaminant removal, contributing to agro-industrial waste valorization and the development of water treatment technologies aligned with the SDGs.

## Graphical abstract

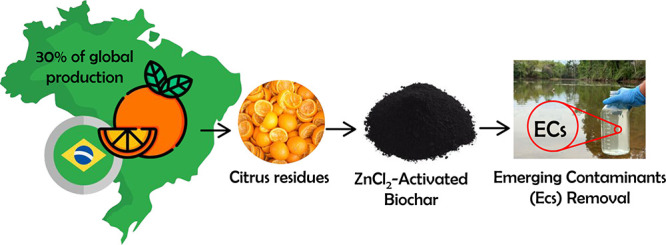

## 1 Introduction

Brazil’s prominent position in the global agro-industrial landscape is consolidated by its world leadership in orange production, with harvests reaching 12.8 million tons.^1^ However, this production generates large volumes of organic waste, including orange peels and bagasse, originating from both the agro-industrial sector and smaller establishments such as restaurants and open-air markets. Although part of this residual biomass is applied in essential oil extraction, composting, animal feed, enzyme production, or as organic fertilizer, its broader use is still limited.^2–4^ This underutilization poses a significant environmental challenge due to rapid fermentation and high organic load.^4^ In this context, interest in sustainable technologies that promote the valorization of residual biomass by converting it into higher value-added products has intensified. Within this framework, the development of adsorbent materials for contaminant removal emerges as a promising alternative.

The control of contamination of water bodies by emerging contaminants (ECs) requires the adoption of environmental remediation strategies.^5,6^ These compounds, which include pharmaceuticals and pesticides, are frequently released into surface and groundwater through unregulated effluents.^7^ Due to their chemical complexity, high persistence, and low biodegradability, ECs are notably refractory to conventional wastewater treatment systems,^8^ and their presence in the environment, even at very low concentrations, is associated with toxic effects and bioaccumulation potential.^5^ Among ECs, pharmaceuticals and particularly antibiotics constitute a subgroup of growing scientific and public health concern,^8^ since their continuous release into the aquatic environment directly contributes to the selection, induction, and dissemination of resistant microorganisms.^9^

Among emerging contaminants, enrofloxacin (ENR) stands out as a representative antibiotic. This fluoroquinolone is widely used in veterinary medicine, frequently detected in surface waters, and exhibits high persistence and potential toxicity.^10^ The removal of fluoroquinolones, such as enrofloxacin (ENR) and ciprofloxacin, remains challenging and represents a growing environmental concern due to their high chemical stability and low rates of natural degradation.^11,12^ This challenge highlights the need for the development of novel, sustainable adsorbent materials for aquatic environments.^13^

Residues from orange processing emerge as promising precursors for biochar (BC) synthesis due to their abundance and lignocellulosic composition.^14^ Recent literature reports the potential of orange peel-derived biochar for the removal of dyes, such as methylene blue and rhodamine B,^15^ as well as antibiotics, including tetracycline.^16^ Despite these promising results, biochar produced from simply pyrolyzed biomass may be insufficient for efficient antibiotic adsorption, highlighting the need for activation. Among the activation routes, the use of ZnCl_2_ is a promising strategy, proven to be effective in converting various lignocellulosic residues into mesoporous materials with high surface area and well-developed porosity, thereby enhancing contaminant removal efficiency.^4,17,18^

In this context, it is hypothesized that ZnCl_2_ activation of orange peel and bagasse residues leads to the formation of a porous biochar with enhanced surface functionality, enabling high adsorption capacity for enrofloxacin through multiple interaction mechanisms. It is also hypothesized that the resulting material maintains its adsorption performance in complex environmental matrices, such as lagoon water, due to its structural and chemical stability. Based on this hypothesis, this work reports the synthesis, characterization, and application of a ZnCl_2_-activated biochar derived from combined citrus residues for the removal of enrofloxacin from aqueous solutions and lagoon water. The study focuses on adsorption performance and mechanism elucidation under both model and real environmental conditions.

## 2 Materials and Methods

### 2.1 Standards and Reagents

All chemicals used in this work were of analytical grade. Enrofloxacin (ENR, CAS 93106-60-6, >98%), amoxicillin (AMOX, CAS 26787-78-0, ≥98%), ceftriaxone (CEFT, CAS 73384-59-5, ≥98%), and 17α-ethinylestradiol (ETHY, CAS 57-63-6, >99.3%) standards, as well as acetonitrile (CAS 75-05-8, 99.99%), acetic acid (CAS 64-19-7, ≥99.7%), and ethanol (CAS 64-17-5, >99.8%), were purchased from Sigma-Aldrich and used as received. Hydrochloric acid (HCl, 37%, CAS 7647-01-0) and sodium hydroxide (NaOH, 99%, CAS 1310-73-2) were acquired from Vetec.

Stock solutions (1000 mg L^–1^) of the ECs were prepared. The ENR stock solution was prepared in acetonitrile containing 0.1% (v/v) acetic acid, whereas the AMOX solution was prepared in ultrapure water obtained from a Milli-Q system (Milli-Q Reference, Millipak) with the addition of 0.1% (v/v) acetic acid. The CEFT solution was also prepared in ultrapure water, and the 17α-ethinylestradiol (ETHY) solution was prepared in ethanol.

Working solutions were obtained by appropriate dilution of the stock solutions to a final concentration of 50 mg L^–1^. The pH of all solutions was adjusted to 4.00 using HCl (0.01 mol L^–1^).

### 2.2 Biochar Synthesis

Orange peel and bagasse residues were collected from commercial establishments in Viçosa, MG, Brazil, and treated as a single mixed biomass matrix, without prior separation or control of the individual fractions. This approach was intentionally adopted to better represent the real composition of agro-industrial waste generated in practice. After collection, the material was thoroughly homogenized before the hydrothermal process to ensure experimental reproducibility and consistency among batches. The material was subsequently homogenized, washed with running water to remove impurities, and dried in an oven at 105 °C for 24 h. After dehydration, the material was ground and sieved through a 35-mesh sieve to obtain a uniform particle size and then stored at room temperature (25 °C).

The synthesis of the activated BC was carried out according to the procedure described by Superbi de Sousa et al.^19^ For this purpose, the biomass was impregnated with ZnCl_2_ at a 1:3 (w/w) ratio in an aqueous medium. The mixture was dried at 105 °C and subsequently pyrolyzed in a muffle furnace using properly covered 150 mL porcelain crucibles, with a heating rate of 10 °C min^–1^ up to 600 °C, which was maintained for 1 h. The resulting material was washed with an HCl solution (0.1 mol L^–1^), followed by rinsing with heated deionized water (80 °C) until neutral pH was achieved. The purified BC was dried at 60 °C for 48 h. After the purification and drying steps, the final biochar yield was determined to be approximately 34%.

### 2.3 Characterizations of Biochar (BC) and Biomass (BM)

The characterization of the BC and precursor biomass (BM) was conducted in accordance with ASTM D 1762-84 (2021) to determine moisture, volatile matter, ash content, and fixed carbon. Elemental CHNS analysis was performed using a LECO TruSPec Micro elemental analyzer following ASTM D 5373-21, while the oxygen content was calculated by difference using eq 1.

%O=100%−(%C+%H+%N+%S+%ash) 1

The thermal behavior of the BC and BM samples was investigated by thermogravimetric analysis (TGA) using a DTG-60H instrument (Shimadzu). The analysis was carried out under an inert nitrogen (N_2_) atmosphere with a flow rate of 50 mL min^–1^. Prior to the analysis, the samples were dried in an oven at 105 °C for 24 h. The TGA analysis consisted of heating the samples from 35 to 800 °C at a heating rate of 10 °C min^–1^.

The morphology of BC and BM was evaluated using a scanning electron microscope (SEM), model JSM-6010LA (JEOL), operated at an accelerating voltage of 20 kV. The samples were mounted on carbon tape and subsequently coated with a thin gold layer using a Quorum Q150R S sputter coater.

The structural characterization of BC and BM was performed by X-ray diffraction (XRD) using a Bruker D* Discover Diffractometer. The analyses were conducted using Cu-Kα radiation with a wavelength of 0.1541 nm, operating in an angular scan (2θ) range from 10° to 50°.

The textural properties of BC and BM were evaluated by nitrogen adsorption–desorption isotherms using a Nova 600 Series analyzer (Anton Paar). The samples, with a particle size of 35 mesh, were subjected to thermal pretreatment to remove moisture and adsorbed gases. The biomass was degassed at 120 °C for 4 h, while the BC was degassed at 220 °C for the same period. The specific surface area was determined by the BET (Brunauer–Emmett–Teller) method, and the pore size distribution was analyzed based on the BJH (Barrett–Joyner–Halenda) model.

The point of zero charge (pH_PZC_) of the materials was determined according to a methodology adapted from ref 20. For this purpose, 15 mg of BC was added to 15.00 mL of NaCl solution (0.100 mol L^–1^), with the initial pH adjusted between 2 and 10. The suspensions were stirred at 180 rpm for 24 h, and the final pH was then measured. The pH_PZC_ was defined as the point at which ΔpH (pH_final_ – pH_initial_) = 0.

To evaluate the surface charge, suspensions containing 10 mg of biochar in 250 mL of of NaCl solution (0.100 mol L^–1^) were prepared and individually adjusted to pH values of 2, 4, 6, 8, 10, and 12. Each suspension was subjected to stirring and sonication, and the zeta potential was determined using a Nano ZS Zetasizer (Anton Paar).

### 2.4 Adsorption Assays

For the adsorption experiments, 20.00 mL of the working solution were transferred to Erlenmeyer flasks. Subsequently, 20 mg of BC were added, and the flasks were kept under agitation at 180 rpm for 24 h at 25 ± 2 °C. Control experiments were conducted under the same conditions, except for the addition of BC. Subsequently, the samples were filtered through cellulose acetate membranes (0.45 μm) and analyzed by UV–vis spectrophotometry (GENESYS 50, Thermo Scientific), using quartz cuvettes. The percentage of adsorption was calculated using eq 2.

$$\mathrm{adsorption}\;(\%)=\left(\frac{C_{i}-C_{e}}{C_{i}}\right)\times 100$$ 2

where *C*_i_ is the initial concentration of the pharmaceutical and *C*_e_ is the equilibrium concentration after adsorption.

Residual ENR concentrations were determined using an analytical calibration curve constructed from standard solutions in the range of 0.5–75 mg L^–1^, analyzed in duplicate. The limits of detection (LOD) and quantification (LOQ) were calculated based on this analytical curve.

#### 2.4.1 Kinetic Study

Adsorption kinetics were evaluated using 15 mg of BC and 15.00 mL of an ENR solution (500 mg L^–1^). The suspensions were agitated at 180 rpm and 25 °C for 24 h. Aliquots were withdrawn at 0, 5, 10, 15, 20, 30, 60, 90, 120, 180, 240, 360, 480, 600, 1140, and 1440 min. The collected samples were subsequently filtered through 0.45 μm cellulose acetate membranes and analyzed by UV–vis spectrophotometry.

The pseudo-first order (eq 3), pseudo-second order (eq 4), and Elovich (eq 5) models were fitted to the experimental data to describe the kinetic behavior of the adsorption process.

$$q_{t}=q_{e}(1-e^{-k_{1}t})$$ 3

$$q_{t}=\frac{k{q_{e}}^{2}t}{1+k_{2}q_{e}t}$$ 4

$$q_{t}=\frac{1}{b}\;\ln(1+\mathrm{abt})$$ 5

In the adsorption process, *q_t_* represents the amount of solute retained per unit mass of the adsorbent at a given time (μg mg^–1^), while *q*_e_ corresponds to the amount adsorbed at equilibrium (μg mg^–1^). The kinetic constant of the pseudo-first order (PPO) model is denoted by *k*_1_ (h^–1^), and that of the pseudo-second order (PSO) model by *k*_2_ (mg μg^–1^ h^–1^). In addition, the parameter a (mg g^–1^ min^–1^) expresses the initial adsorption rate, while *b* (mg g^–1^) is associated with the desorption constant. The experimental time is denoted by *t* (h).

#### 2.4.2 Influence of pH on the Adsorption Performance

The pH of the ENR solution (500 mg L^–1^) was adjusted to 4, 6, 7, 8, and 10 using HCl or NaOH solutions (both at 0.100 mol L^–1^). Subsequently, 15 mg of biochar were added, and the suspensions were agitated for 4 h at 25 °C. After filtration, the residual ENR concentration was determined by UV–vis spectrophotometry.

#### 2.4.3 Adsorption Isotherm

Adsorption isotherms were obtained using 15 mg of biochar in 15.00 mL of ENR solutions with concentrations ranging from 75 to 500 mg L^–1^. These concentrations were intentionally selected to promote saturation of the adsorption sites, enabling reliable isotherm modeling and accurate determination of the maximum adsorption capacity (*q*_max_), which is essential for evaluating and comparing the performance of the adsorbent. The suspensions were maintained under agitation at 180 rpm for 4 h at 25 °C. After equilibrium was reached, the samples were centrifuged and filtered through 0.45 μm membranes, and the residual concentration was determined by UV–vis spectrophotometry.

#### 2.4.4 Reusability and Regeneration of Biochar

After each adsorption cycle, the biochar was recovered by centrifugation at 4000 rpm and subsequently regenerated. Reuse experiments were performed employing different desorbing solutions. Desorption was carried out using either 10 mL of ethanol or 10 mL of NaOH solution (0.1 mol L^–1^) under agitation (180 rpm) for 30 min at 25 °C. Subsequently, the material was washed with 10 mL of type II water under agitation for 10 min, centrifuged, dried, and reused in the subsequent adsorption cycle. The adsorption–desorption procedure was repeated for up to consecutive cycles.

#### 2.4.5 Evaluation of Interferences in the Removal of ENR by BC

To evaluate the influence of interferents, an ENR solution (500 mg L^–1^) was prepared using water from the lake at the Universidade Federal de Viçosa, Viçosa, Minas Gerais-Brazil (−20.765935, −42.869956), collected on March 18, 2025, at 9:00 am (Figure S1). The electrical conductivity was determined using an AZ 86503 conductivity meter. The remaining analyses were performed at the Water Quality Control Laboratory (LCQA) of the local water treatment plant, and the main physicochemical characteristics were determined according to the methods described in Table 1. Adsorption experiments were carried out using 15 mg of biochar in 15.00 mL of the solution under agitation (180 rpm) for 4 h at controlled temperature. The samples were filtered (0.45 μm), and the residual enrofloxacin concentration was determined by UV–vis spectrophotometry.

**Table 1:** Physicochemical Analyses of the Pond Water Sample Collected in Viçosa, Minas Gerais, Brazil (−20.765935, −42.869956)

parameter	method^a^
pH	SMEWW 4500-H+ B
turbidity (NTU)	SMEWW 2130 B
apparent color/uC	SMEWW 2120 E
total alkalinity	SMEWW 2320
chlorophyll-*a*	SMEWW 10200 H
total phosphorus	SMEWW 4500-P D
ammoniacal nitrogen	HACH 8038
dissolved oxygen (DO)	SMEWW 5210 B

a SMEWW—Standard Methods of Examination of Water and Wastewater, 24 rd ed. 2022.

#### 2.4.6 Evaluation of Zn Leaching

Inductively coupled plasma optical emission spectroscopy (ICP-OES) was employed to evaluate the stability of residual Zn remaining after chemical activation. Biochar samples (15 mg) were placed in contact with 15.00 mL of aqueous solution under agitation (180 rpm) for 4 h at 25 °C. After filtration through a 0.45 μm membrane, the supernatant was analyzed by ICP-OES using an iCAP PRO instrument (Thermo Scientific, USA), monitoring the Zn emission line at 206 nm. All measurements were performed in duplicate.

#### 2.4.7 Statistical Analysis

All experiments were performed at least in duplicate, and the results are presented as mean ± standard deviation. Data treatment and calculations were performed using Microsoft Excel 2019 (Microsoft Corporation, USA), while graphs and data processing were carried out using OriginPro 2024 (OriginLab Corporation, USA). Model fitting was evaluated using the correlation coefficient (*R* ^2^) and the Akaike Information Criterion (AIC), when applicable.

## 3 Results and Discussion

### 3.1 Characterization of Materials

The materials were subjected to immediate analysis, and the results are presented in Table 2. A significant reduction in volatile matter content was observed, decreasing from 87.28% in the raw biomass to 22.16% in the biochar.

**Table 2:** Proximate Analysis of Orange Peel and Bagasse Biomass (BM) and ZnCl_2_-Activated Biochar (BC)

material	humidity (%)	volatile content (%)	ash content (%)	fixed carbon (%)
BM	10.24	87.28	3.48	9.24
BC^a^	24.63	22.16	14.74	63.10

a BC: produced at 600 °C for 1 h using ZnCl_2_ at a 1:3 (w/w) ratio relative to the BM.

Conversely, the fixed carbon content increased from 9.24 to 63.10%, while the ash content rose from 3.48 to 14.74%. These changes indicate the removal of volatile organic compounds and the concentration of carbon during the pyrolysis process, resulting in a more stable and carbon-rich material, with a profile similar to that reported in other studies on biochars produced under comparable conditions.^21,22^ In addition, the increase in ash content reflects the concentration of nonvolatile minerals remaining after the thermal process.

The results of the elemental chemical composition of the raw biomass and the biochar are presented in Table 3. The analysis reveals significant changes in the elemental composition of the material after the pyrolysis process. A pronounced increase in carbon content was observed, rising from 41.26% in the biomass to 59.91% in the biochar. In contrast, the hydrogen and oxygen contents decreased from 6.49 and 45.39% in BM to 2.53 and 18.99% in BC, respectively. Sulfur, present in the raw material at 2.36%, was not detected (ND) in the biochar, indicating its complete volatilization during thermal treatment.

**Table 3:** Elemental Analysis (C, H, N, S, O) and Atomic Ratios (H/C and O/C) of Orange Peel and Bagasse Biomass (BM) and ZnCl_2_-Activated Biochar (BC)

material	C	H	N	S	O^c^	H/C	O/C
BM	41.26	6.49	1.0	2.36	45.39	1.87	0.83
BC^a^	59.91	2.53	3.8	ND^b^	18.99	0.50	0.24

a BC: produced at 600 °C for 1 h using ZnCl_2_ at a 1:3 (w/w) ratio relative to the biomass (BM).

b ND: not detected.

c O calculated by difference: %O = 100% – (%C + %N + %H + %S + %ash).

The atomic H/C and O/C ratios, frequently used to assess the degree of pyrolysis, provide a clear insight into the chemical transformations that occur during the process. It can be observed that the biomass exhibits higher H/C (1.87) and O/C (0.83) values, reflecting its lignocellulosic nature and the presence of hydrophilic functional groups in the original material.^18^ After pyrolysis, the biochar exhibited a reduction in these ratios, reaching 0.50 for H/C and 0.24 for O/C. The decrease in the H/C ratio is characteristic of dehydration (loss of H_2_O) and demethylation (loss of CH_4_) processes, which remove hydrogen from the structure and increase its aromaticity.^23^ This transformation indicates that the biochar has a more stable structure, with a higher degree of aromaticity and lower polarity compared to the precursor biomass. The atomic H/C ratio of 0.50 obtained for the biochar derived from orange peel and pulp falls within a range characteristic of carbonaceous materials with a high degree of aromatic condensation, corroborating the patterns of thermal stability observed by the research group for complex matrices. Similar results were reported by Silva et al. for biochar produced from malt residue.^18^ Likewise, biochar derived from industrial biological sludge also showed comparable behavior, as reported by Netto et al.^24^ These findings demonstrate that the pyrolysis process effectively consolidates a robust adsorbent matrix, regardless of the origin of the precursor biomass.

The thermal degradation profiles obtained by thermogravimetric analysis (TGA) and its derivative (DTG) for the biomass and the biochar are presented in Figure S2. Initially, a mass loss event common to both materials is observed in the temperature range of 25–150 °C. This stage is associated with the volatilization of physically adsorbed moisture or free water, a typical behavior of materials of vegetal origin. In the biomass (Figure S2a), several thermal events are observed, in contrast to the BC (Figure S2b), with multiple events identified across the temperature range from 150 to 600 °C. In addition, mass loss events occurring at relatively low temperatures (below 250 °C) can be attributed to the thermal degradation of extractives, such as essential oils, soluble sugars, and organic acids, which are typically abundant in citrus residues and contribute significantly to the early release of volatile compounds.^25^ It is observed that, in the DTG curve of the biomass, the peaks associated with the degradation regions are significantly more pronounced compared to those of the biochar. Such events can be attributed to the thermal decomposition of the main lignocellulosic polymers. Hemicelluloses, due to their amorphous and branched structure, exhibit lower thermal stability and undergo primary degradation between 200 and 320 °C.^14^ The thermal decomposition of cellulose occurs in two main stages: the amorphous fraction, which is less structurally ordered, degrades at lower temperatures (≈250–320 °C), whereas the crystalline fraction, which requires higher activation energy, is responsible for the intense mass loss observed in the 300–400 °C range.^26^ Finally, lignin exhibits a slow and continuous decomposition process, extending from moderate temperatures to above 600 °C, a behavior associated with the complexity of its aromatic rings and cross-linked structures.^14^ These results indicate that the biochar exhibits higher thermal stability compared to the precursor biomass. This difference is associated with the higher presence of volatile organic compounds and noncarbonized material in the raw biomass. Such behavior is consistent with the FTIR observations (Figure 1a), which confirmed the greater abundance of organic functional groups in the biomass structure.

**Figure 1:**
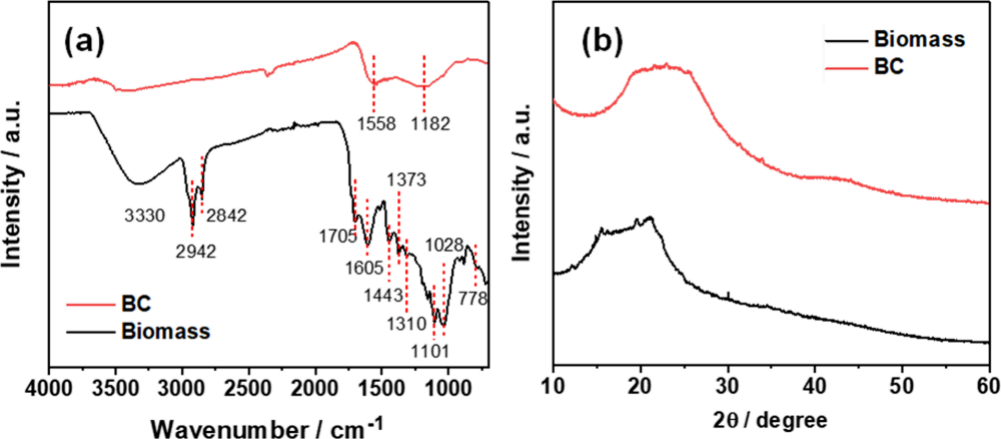
Characterization of orange peel and bagasse biomass (BM) and ZnCl_2_-activated biochar (BC) by (a) Fourier transform infrared spectroscopy with attenuated total reflectance (FTIR-ATR) and (b) X-ray diffraction (XRD). BC: produced at 600 °C for 1 h using ZnCl_2_ at a 1:3 (w/w) ratio relative to the BM.

In the FTIR spectra, the biomass exhibits a broad and intense band in the 3419–3523 cm^–1^ region, attributed to the ν­(O–H) stretching vibration, characteristic of hydroxyl groups present in cellulose, lignin, and pectin, which are the major components of orange peel.^23,27^ The bands at 2942 and 2842 cm^–1^ can be attributed to the asymmetric and symmetric ν­(CH) stretching vibrations of methyl and methylene groups in aliphatic chains, whose presence is typical of raw biomass.^28^ The band at 1705 cm^–1^ can be related to the ν­(C=O) stretching vibration of carbonyl groups in ketones, esters, and carboxylic acids, mainly associated with the hemicellulose and pectin fractions.^22,29^ The band at 1605 cm^–1^ can be associated with skeletal vibrations of the lignin aromatic ring, as well as with adsorbed water.^30^ The band at 1443 cm^–1^ corresponds to the angular deformations δ­(CH_2_) and δ_as_(CH_3_), while the absorption at 1373 cm^–1^ is related to the symmetric deformation δ­(CH) of cellulose and hemicelulose.^29^ The intense bands at 1101 and 1028 cm^–1^ are characteristic of the ν­(C–O–C) and ν­(C–O) stretching vibrations of primary and secondary alcohols, confirming the polysaccharide structure of the precursor biomass.^22^ The band at 778 cm^–1^ refers to the out-of-plane γ­(C–H) deformation of aromatic rings, indicative of the presence of lignin units.^29^ After the pyrolysis process, a significant reduction in the intensity or even the disappearance of oxygenated (O–H and C–O) and aliphatic functional bands is observed, resulting from dehydration and volatilization of organic matter, a behavior commonly reported for biochars derived from citrus residues.^23^ On the other hand, a band at 1558 cm^–1^ is observed, attributed to the C=C stretching vibrations of aromatic rings, indicating an increase in aromaticity and the formation of graphitic structures.^24^ The band at 1182 cm^–1^ suggests the presence of C–O stretching vibrations of phenolic or ester groups.^29^

The materials were characterized by X-ray diffraction (XRD), as shown in Figure 1b. The biomass exhibited a broad peak in the 2θ range of 10–30°, associated with the semicrystalline nature of cellulose and hemicelluloses. In the case of the biochar, a broad halo with a slight shift toward higher angles (to the right) was observed, which can be related to the pyrolysis process, resulting from the removal of organic constituents and structural reorganization during pyrolysis. Similar results were reported by Gonzalez-Canche et al.^31^

The materials were also characterized by N_2_ physisorption (Figure 2). While the precursor biomass exhibited a type II isotherm, typical of nonporous or macroporous materials, the activated biochar displayed type IV isotherms, consistent with the structure of mesoporous materials.^32^ The corresponding textural and structural properties obtained from the N_2_ physisorption analysis are summarized in Table S1.

**Figure 2:**
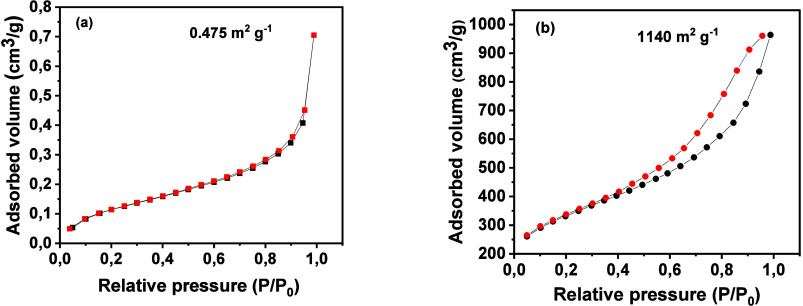
N_2_ adsorption–desorption isotherms of (a) orange peel and bagasse biomass (BM) and (b) ZnCl_2_-activated biochar. BC: produced at 600 °C for 1 h using ZnCl_2_ at a 1:3 (w/w) ratio relative to the biomass (BM).

The raw biomass exhibited a very low specific surface area (0.475 m^2^ g^–1^), negligible micropore volume, and a limited total pore volume, indicating an underdeveloped porous structure with low accessibility. After chemical activation with ZnCl_2_, a remarkable increase in the textural properties was observed, with the surface area reaching 1140 m^2^ g^–1^, accompanied by a micropore volume of 0.1589 cm^3^ g^–1^ and a total pore volume of 1.1862 cm^3^ g^–1^. These results demonstrate the crucial role of ZnCl_2_ as an activating agent in promoting the formation of a highly developed porous network, with contributions from both micropores and mesopores. The significant increase in microporosity is mainly attributed to the dehydrating effect of ZnCl_2_ and its ability to enhance the decomposition of the lignocellulosic matrix during carbonization, favoring pore formation and cavity development.^33^ In addition, the average pore diameter remained within the mesoporous range (2–50 nm), indicating the formation of a hierarchical porous structure that may facilitate molecular diffusion and improve adsorption performance. The high surface area obtained is consistent with values reported in the literature for activated carbons produced from lignocellulosic and agro-industrial residues using ZnCl_2_ as the chemical activating agent. El Nemr et al.^17^ reported an orange peel-derived biochar with a specific surface area of 1228.2 m^2^ g^–1^, while Deshmukh et al.^34^ obtained a lower value of 512.2 m^2^ g^–1^ using a similar precursor. Additionally, Fernandez et al.^15^ achieved a surface area of 1090 m^2^ g^–1^ for orange peel-derived biochar chemically activated with H_3_PO_4_. These findings demonstrate that the textural properties of biochar are strongly influenced by both the precursor composition and the activation method employed.

The SEM images (Figure 3), acquired at the same magnification, allow a direct comparison between the precursor biomass and the activated biochar. The in natura biomass (Figure 3a) exhibits a compact and relatively smooth surface, consistent with its very low specific surface area (<1 m^2^ g^–1^). In contrast, the activated biochar (Figure 3b) presents a markedly altered morphology, characterized by a fragmented, irregular, and rough surface with evident structural disruption. These morphological changes demonstrate the effectiveness of the chemical activation and pyrolysis processes in promoting the development of a more porous and heterogeneous carbonaceous structure.

**Figure 3:**
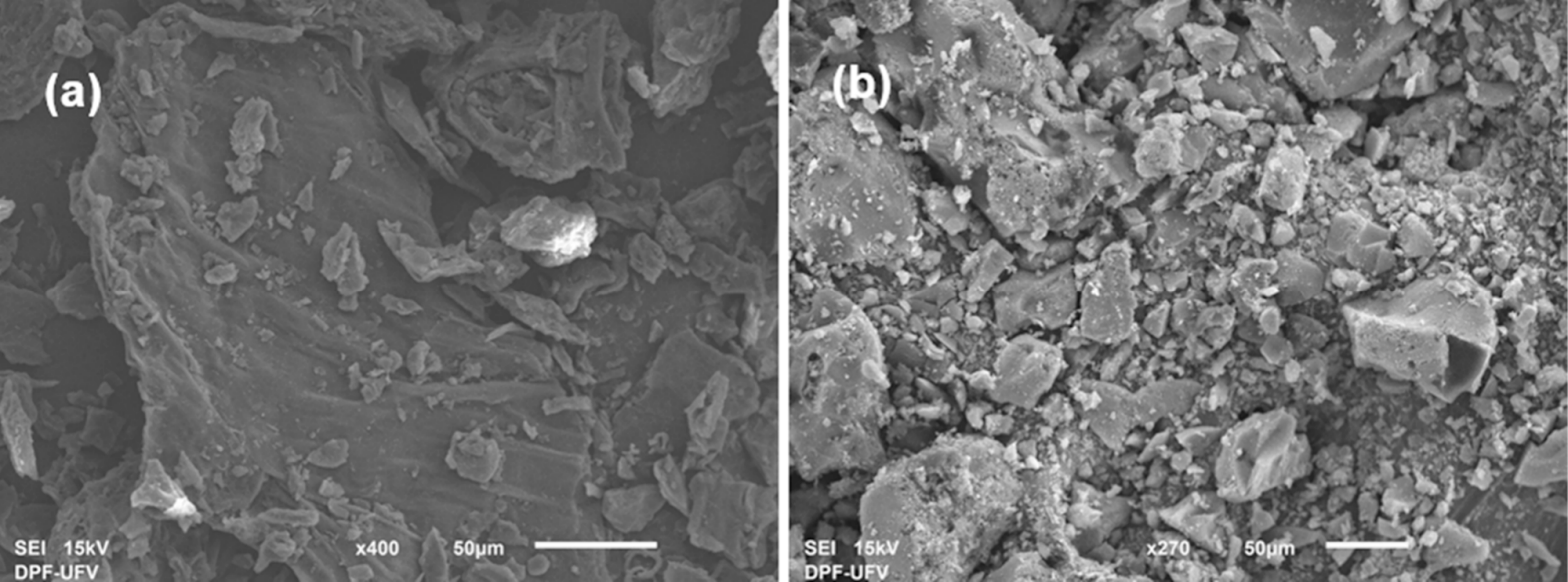
Scanning electron microscopy (SEM) images of (a) orange peel and bagasse biomass (BM) and (b) ZnCl_2_-activated biochar. BC: produced at 600 °C for 1 h using ZnCl_2_ at a 1:3 (w/w) ratio relative to the biomass (BM).

At 600 °C, ZnCl_2_ acts as a chemical activating agent, promoting dehydration and selective volatilization of organic components. The concomitant release of gaseous species during pyrolysis increases the internal pressure within the biomass structure. This pressure exceeds the mechanical resistance of the cell walls, causing their rupture and resulting in a fragmented and rough carbonaceous matrix.^35^ The presence of cavities and irregular channels on the biochar surface not only corroborates the mesoporous nature identified by the adsorption isotherms but also explains the increase in surface area to 1140 m^2^ g^–1^. This rough network facilitates the diffusion and access of contaminants to internal active sites, a morphological behavior characteristic of highly efficient activated carbons.^24^

The point of zero charge (pH_PZC_) of the biochar was determined to be 7.67 (Figure S3). Below this pH value, the surface exhibits a net positive charge, whereas at higher pH values it becomes negatively charged. This slightly alkaline character evidence the chemical modification of the precursor biomass induced by pyrolysis. The process leads to the degradation of acidic functional groups and the formation of basic mineral species, such as carbonates. As a result, the surface pH of the material increases. Similar findings were reported by Afolabi, who observed a transition from the acidic nature of raw orange peel (pH_PZC_ 3.83) to a basic character (pH_PZC_ 10.03) following carbonization at 500 °C. This increase in pH_PZC_ was attributed to the decomposition of organic acids and the volatilization of acidic oxygen-containing functional groups, such as carboxylic and phenolic groups, during thermal treatment.^36^ Additionally, the formation of ash rich in alkali and alkaline earth metals, such as potassium (K) and calcium (Ca), contributes to the surface basicity, as evidenced by Gonzalez-Canche et al.^31^ The results of this work indicate that the biochar surface is amphoteric, promoting electrostatic interactions with both cationic and anionic species, depending on the pH of the medium.

The zeta potential results (Figure S4) indicate that pyrolysis induced significant changes in the material’s surface chemistry. While the precursor biomass exhibited a negative charge at pH 2, the biochar showed a net positive charge (+5.9 mV) under strongly acidic conditions. This behavior is attributed to the protonation of surface functional groups and the loss of acidic groups during carbonization. The isoelectric point of the biochar was determined to be in the pH range of 2–4. This result is consistent with typical values reported for lignocellulosic biochars, where the negative surface charge increases progressively with pH due to the deprotonation of residual carboxylic and phenolic groups.^37^

### 3.2 Adsorption Assays

The adsorption performance of different CECs on the ZnCl_2_-activated biochar was evaluated. As shown in Figure 4, ceftriaxone and enrofloxacin exhibited the highest removal efficiencies, approaching 100%, whereas amoxicillin and ethinylestradiol achieved lower removals of 57 and 25%, respectively.

**Figure 4:**
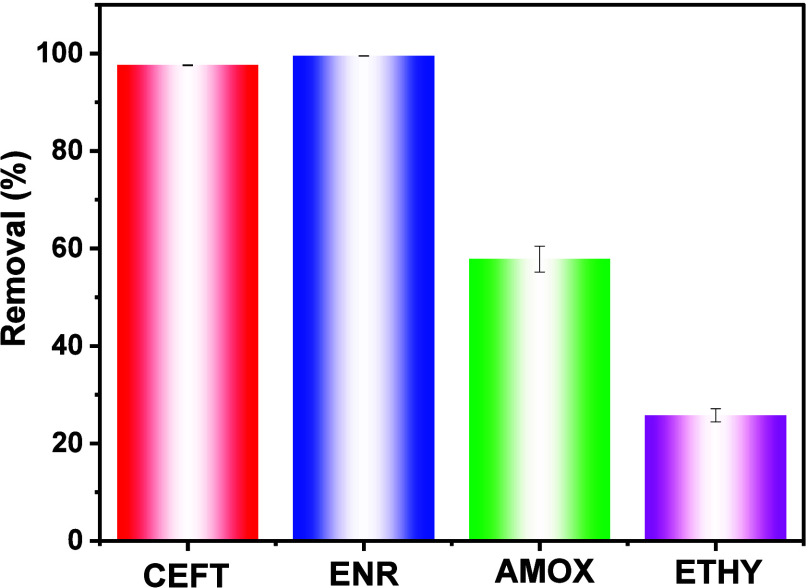
Removal of emerging contaminants (ceftriaxone—CEFT, enrofloxacin—ENR, amoxicillin—AMOX, and ethinylestradiol—ETHY) by ZnCl_2_-activated biochar derived from orange peel and bagasse. Experimental conditions: initial concentration: 50 mg L^–1^, volume: 20 mL, adsorbent mass of 20 mg, pH = 4, agitation: 180 rpm, time: 24 h, temperature: 25 ± 2 °C.

Due to its high removal efficiency and its significance as a fluoroquinolone antibiotic frequently detected in aquatic environments, enrofloxacin was selected for further studies. This allowed a detailed investigation of the adsorption mechanisms and optimization of removal conditions. Adsorption assays were also conducted using the raw biomass; however, upon contact with water, the biomass underwent partial solubilization. This behavior is mainly attributed to the high content of hydrophilic extractives, such as sugars, pectins, and organic acids, characteristic of citrus residues. The release of these compounds increased the solution turbidity, directly interfering with spectrophotometric measurements and compromising data reliability, thus preventing the acquisition of representative results for evaluating adsorption performance.

### 3.3 Adsorption of Enrofloxacin by ZnCl_2_-Activated Biochar

Initially, the analytical parameters for ENR were assessed. The analytical curve (Figure S5) exhibited a linear relationship between concentration and absorbance, with an *R* ^2^ of 0.995. The residuals plot (inset of Figure S5) showed a random distribution around zero, with no systematic trend, confirming the method’s reliability. Using the calibration parameters and the standard deviation of the blank, the limits of detection (LOD) and quantification (LOQ) were determined as 3.18 and 10.62 mg L^–1^, respectively.

The effect of solution pH on ENR adsorption onto the biochar (Figure S6) shows a strong dependence on acid–base conditions, with maximum removal observed at pH 8 (*q*_e_ = 238.02 ± 13.36 mg g^–1^). ENR has two p*K*_a_ values: p*K*_a1_ ≈ 5.32, corresponding to the dissociation of the carboxylic (−COOH) group, and p*K*_a2_ ≈ 8.72, associated with the piperazinyl group.^38^ The nitrogen at the para position of ENR is substituted with an ethyl group, which reduces its basicity and limits protonation under acidic conditions.^39^ Thus, at pH values above 5.32, the molecule predominantly exhibits an anionic character (ENR^–^). Although ENR is anionic at pH values above 5.32, adsorption is maximized at pH 8 due to the balance between the charges of the adsorbent and the molecule. As the pH increases, the zeta potential of the biochar becomes progressively more negative. Near the point of zero charge (pH_PZC_ = 7.7), the surface charge density is still relatively low (−20 mV, Figure S4), which minimizes electrostatic repulsions and favors interaction with ENR, which exhibits a heterogeneous charge distribution, with negatively charged regions and apolar domains (Figure S7). This behavior explains the high removal of a molecule that, although anionic, has only one of its functional groups charged. In addition to electrostatic interactions, hydrogen bonding and π–π interactions also contribute to the adsorption process.^40–42^

The kinetic study of ENR adsorption onto the biochar (Figure 5a) showed a rapid uptake during the initial minutes, approaching equilibrium within 200 min. This fast initial adsorption can be attributed to the high availability of active sites on the biochar surface.^43^ As these higher-energy sites become occupied, the adsorption rate gradually decreases until the system reaches equilibrium, a behavior characteristic of adsorption processes on heterogeneous surfaces. The kinetic data were fitted using different adsorption models. The pseudo-second order (PSO) model did not satisfactorily describe the experimental data, particularly in the initial adsorption stage, where significant deviations associated with pronounced curvature were observed. This behavior suggests that the adsorption process is not governed exclusively by chemisorption and likely involves multiple interaction mechanisms between the adsorbate and the adsorbent surface. In contrast, the Elovich model provided the best fit over the entire investigated time range, indicating adsorption on a heterogeneous surface with energetically distinct active sites. These results also suggest the coexistence of multiple adsorption mechanisms, including surface heterogeneity and diffusion-related effects.^44^ These include electrostatic interactions, hydrogen bonding, and π–π interactions between the aromatic rings of ENR and the carbonaceous matrix of the biochar.^41,42,45^

**Figure 5:**
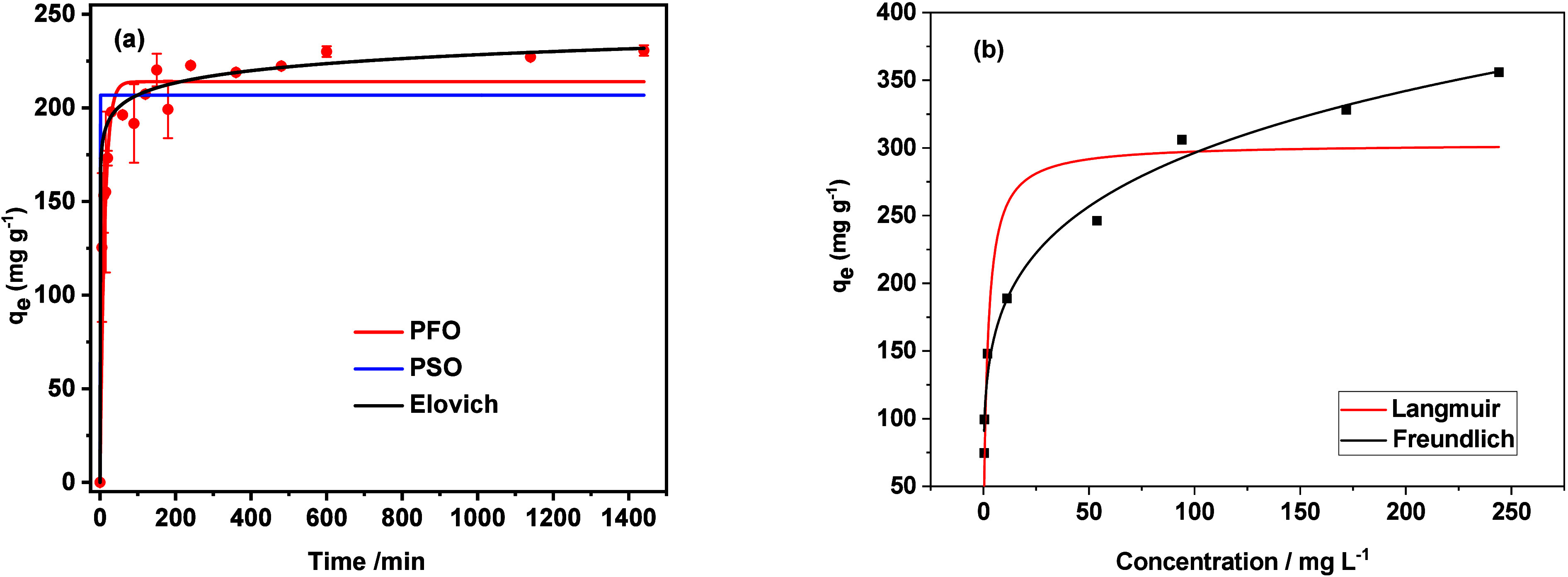
(a) Kinetic study of enrofloxacin (ENR) adsorption onto ZnCl_2_-activated biochar derived from orange peel and bagasse, fitted to the pseudo-first order (PFO), pseudo-second order (PSO), and Elovich models. Experimental conditions: temperature ≈25 °C, pH = 8, initial ENR concentration of 500 mg L^–1^, agitation at 180 rpm, and total contact time of 24 h. (b) Equilibrium adsorption isotherm of enrofloxacin (ENR). Experimental conditions: temperature ≈25 °C, pH = 8, initial ENR concentrations of 75–500 mg L^–1^, agitation at 180 rpm for 4 h.

The adsorption isotherm assays of ENR onto the biochar are shown in Figure 5b. The experimental data were better described by the Freundlich model than by the Langmuir model (Table 4). This indicates that adsorption occurs mainly in multilayers on an energetically heterogeneous surface. No defined saturation plateau was observed within the evaluated concentration range. Such behavior reflects the heterogeneity of adsorption sites, which governs the ENR removal capacity within the biochar pore structure.

**Table 4:** Isotherm Parameters for Enrofloxacin Adsorption on ZnCl_2_-Activated Biochar Derived from Orange Peel and Bagasse^a^

model	parameters	value
Langmuir	*q*_max_ (mg g^–1^)	303.08 ± 23.93
	*K*_L_ (L mg^–1^)	0.5121 ± 0.2568
	*R* ^2^	0.8343
	AIC	71.30566
Freundlich	*K*_F_ (mg g^–1^)/(mg L^–1^)1/*n*	114.21 ± 6.33
	*n*	4.82 ± 0.28
	*R* ^2^	0.9879
	AIC	50.32322

a BC: produced at 600 °C for 1 h using ZnCl_2_ at a 1:3 (w/w) ratio relative to the biomass (BM).

ZnCl_2_-activated biochar from orange peel and bagasse was compared with biochars from other biomasses for enrofloxacin removal (Table 4). Notably, biochars from spent malt bagasse were produced via hydrothermal carbonization (150 °C, H_3_PO_4_) and pyrolysis (500 °C). The hydrothermal biochar showed the highest adsorption capacity (164 mg g^–1^), while pyrolyzed materials were less effective.^38^ Other biochars from different biomasses, including biological sludge from the cosmetics industry and bamboo sawdust, were also tested for enrofloxacin removal, showing much lower maximum adsorption capacities of 15.83 and 45.88 mg g^–1^, respectively.^28,46^ In this work, ZnCl_2_-activated biochar from orange peel and bagasse showed superior performance for enrofloxacin removal, reaching a maximum adsorption capacity (*q*_max_) of 238.02 mg g^–1^, higher than values reported for biochars from other biomasses. These results underscore the potential of orange peel and bagasse as precursors for efficient adsorbents and confirm the effectiveness of ZnCl_2_ activation for removing this antibiotic.

The reusability and stability of the ZnCl_2_-activated biochar were evaluated through consecutive adsorption–desorption cycles using two different desorbing agents. As shown in Figure 6, when regenerated with ethanol, the biochar maintained excellent adsorption performance throughout the reuse cycles, exhibiting removal efficiencies close to 100 mg g^–1^ over all four cycles. For NaOH, a slight decrease in adsorption capacity was observed in the second cycle (73.36 ± 1.32 mg g^–1^), followed by recovery in the subsequent cycles (90.40 ± 1.03 and 91.87 ± 1.27 mg g^–1^). Similar behavior was reported by Netto et al.,^47^ who observed an initial decrease in adsorption efficiency after regeneration with NaOH, attributed to surface modifications caused by the alkaline medium. The recovery observed in the later cycles may be associated with the reactivation of previously blocked adsorption sites or with stabilization of the material surface after the first regeneration step.

**Figure 6:**
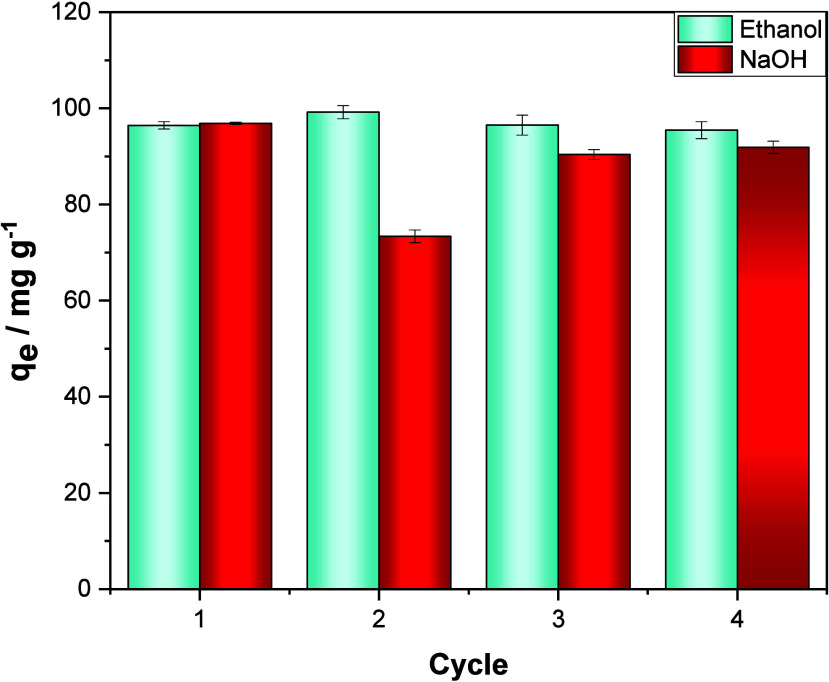
Reusability performance of the ZnCl_2_-activated biochar over consecutive adsorption–desorption cycles using different desorbing agents. Conditions: 15 mg of biochar and 15 mL of ENR solution (100 mg L^–1^) at 25 °C for 4 h under agitation (180 rpm). Desorption was performed using 10 mL of ethanol or NaOH solution (0.1 mol L^–1^) for 30 min at 25 °C. Error bars represent the standard deviation of duplicate experiments.

To assess practical applicability, the biochar was evaluated in lake water from the Universidade Federal de Viçosa, spiked with enrofloxacin. The aqueous matrix was first characterized (Table 5). The pond water had an electrical conductivity of 79.5 μS cm^–1^, compared to 0.98 μS cm^–1^ for distilled water, indicating higher ionic strength and more dissolved solids. Other parameters included alkalinity of 32.93 mg L^–1^, chlorophyll-*a* of 9.9 μg L^–1^, COD of 17 mg L^–1^, and pH 7.69. These values describe a moderately complex matrix that can compete for the adsorbent’s active sites. Despite these interferents, the biochar maintained high adsorption performance. In pond water (Table 5), the equilibrium adsorption capacity (*q*_e_) reached 223.67 ± 2.14 mg g^–1^, demonstrating the adsorbent’s robustness and confirming its potential for tertiary effluent treatment.

**Table 5:** Evaluation of the Effect of Potential Interferents on the Removal of Enrofloxacin (ENR) by Biochar Derived from Orange Peel and Bagasse

biochar	conductance (μs)	*q*_e_ (mg g^–1^)
lake water^a^	79.5	223.67 ± 2.14
type I water	0.98	234.49 ± 3.61

a Lake water from the Universidade Federal de Viçosa, Viçosa, Minas Gerais, Brazil (−20.765935, −42.869956), collected on 18 March 2025 at 9:00 am (Figure S1).

Leaching assays indicated an approximate Zn loss of 4.5%, demonstrating that a small fraction of the metal remains susceptible to release into the aqueous phase even after the washing step performed during synthesis. This behavior is consistent with previous studies on ZnCl_2_-activated biochars, in which residual Zn species remain partially incorporated into the carbon matrix or associated with oxygen-containing functional groups, and may be gradually released in aqueous solution.^48^ Recent studies have reported that ZnCl_2_ activation promotes the formation of Zn–O bonds and the partial stabilization of Zn species within the carbon structure, explaining the residual leaching observed.^48^

## 4 Conclusion

ZnCl_2_-activated biochar derived from orange residues exhibited favorable physicochemical properties and high efficiency in enrofloxacin removal, demonstrating the effectiveness of chemical activation in enhancing surface area, porosity, and the availability of active functional groups. Adsorption was favored at near-neutral pH (≈8). In addition to electrostatic interactions, other mechanisms, including hydrogen bonding, π–π interactions, and hydrophobic interactions, likely contribute to the adsorption process. The adsorption kinetics were well described by the Elovich model, while the Freundlich isotherm indicated surface heterogeneity. The material’s robustness in the presence of interferents in natural water supports its practical applicability in real water treatment scenarios. Overall, this study advances the valorization of agro-industrial residues and identifies ZnCl_2_-activated orange biochar as a promising and sustainable option for the removal of emerging contaminants, particularly environmentally persistent antibiotics such as enrofloxacin.

## Supplementary Material



## Data Availability

All data are availabel in the text.

## Notes

Lagoon water sample collected in Viçosa, Minas Gerais, Brazil (−20.765935, −42.869956) (Figure S1); thermogravimetric analysis (TGA/DTG) of (a) orange peel and bagasse biomass (BM) and (b) ZnCl_2_-activated biochar; BC: produced at 600 °C for 1 h using ZnCl_2_ at a 1:3 (w/w) ratio relative to the biomass (BM) (Figure S2); pH at the point of zero charge (pH_PZC_) of ZnCl_2_-activated biochar (BC); BC: produced at 600 °C for 1 h using ZnCl_2_ at a 1:3 (w/w) ratio relative to the biomass (Figure S3); zeta potential of ZnCl_2_-activated biochar (BC) and orange peel and bagasse biomass (BM); BC: produced at 600 °C for 1 h using ZnCl_2_ at a 1:3 (w/w) ratio relative to the BM (Figure S4); analytical curve of enrofloxacin over the concentration range of 0.5–75 mg L^–1^ (Figure S5); effect of pH on the adsorption capacity of enrofloxacin (ENR) by ZnCl_2_-activated biochar; experimental conditions: initial ENR concentration of 500 mg L^–1^, adsorbent mass of 15 mg, and agitation for 4 h at 25 °C (Figure S6); and van der Waals surface colored by electrostatic potential of enrofloxacin; adapted from ref 38 with permission from Elsevier (Figure S7) (PDF)
