# Supplementary material for: Upcycling Orange-Derived Peel and Bagasse into ZnCl2‑Activated Biochar for Sustainable Enrofloxacin Removal
Source: ACS Omega. 2026 Jun 26;11(27):39891–901. doi: 10.1021/acsomega.6c00992 (PMC13382840; doi:10.1021/acsomega.6c00992)
Supplement: Supplementary file 1 [file ao6c00992_si_001.pdf]

Supporting Information

Upcycling Orange-Derived Peel and Bagasse into $ZnCl_2$-Activated Biochar for Sustainable Enrofloxacin Removal

Kleryton Luiz Alves de Oliveira[1], Ana Luíza Ferreira de Matos[1], Marcela de Oliveira Brahim Cortez[1], Antonio Machado Netto[1], Noemí Cristina Silva de Souza[1], Angélica de Cassia Oliveira Carneiro[2] and Renata Pereira Lopes Moreira[1*]

[1]Department of Chemistry, Universidade Federal de Viçosa, Peter Henry Rolfs Avenue, University Campus (no number), Viçosa 36570-900, MG, Brazil.

[2]Department of Forest Engineering, Universidade Federal de Viçosa, Peter Henry Rolfs Avenue, University Campus (no number), Viçosa 36570-900, MG, Brazil.

Corresponding Author

Renata Pereira Lopes Moreira

Department of Chemistry

Universidade Federal de Viçosa

Peter Henry Rolfs Avenue, University Campus

Viçosa, MG 36570-900, Brazil

E-mail: renata.plopes@ufv.br

Table of Contents

Figure S1. Lagoon water sample collected in Viçosa, Minas Gerais, Brazil (−20.765935, −42.869956). 3

Figure S2. Thermogravimetric analysis (TGA/DTG) of (a) orange peel and bagasse biomass (BM) and (b) $ZnCl_2$-activated biochar. BC: produced at 600 °C for 1 h using $ZnCl_2$ at a 1:3 (w/w) ratio relative to the biomass (BM). 3

Figure S3. pH at the point of zero charge (pH_{PZC}) of $ZnCl_2$-activated biochar (BC). BC: produced at 600 °C for 1 h using $ZnCl_2$ at a 1:3 (w/w) ratio relative to the biomass. 4

Figure S4. Zeta Potential of $ZnCl_2$-activated biochar (BC) and orange peel and bagasse biomass (BM). BC: produced at 600 °C for 1 h using $ZnCl_2$ at a 1:3 (w/w) ratio relative to the BM 4

Figure S5. Analytical curve of enrofloxacin over the concentration range of 0.5–75 mg L^{-1}. 5

Figure S6. Effect of pH on the adsorption capacity of enrofloxacin (ENR) by $ZnCl_2$-activated biochar. Experimental conditions: initial ENR concentration of 500 mg L^{-1}, adsorbent mass of 15 mg, agitation for 4 h at 25 °C. 5

Figure S7. Van der Waals surface colored by electrostatic potential of enrofloxacin. Adapted from Ref. [1] with permission from Elsevier. 6

Table S1. Textural Properties of Biomass and $ZnCl_2$-Activated Biochar 6

Reference 6

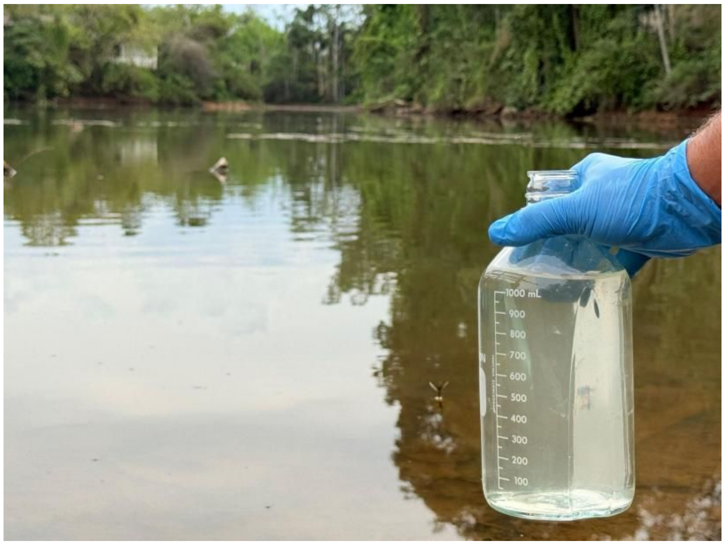

Figure S1. Lagoon water sample collected in Viçosa, Minas Gerais, Brazil (−20.765935, −42.869956).

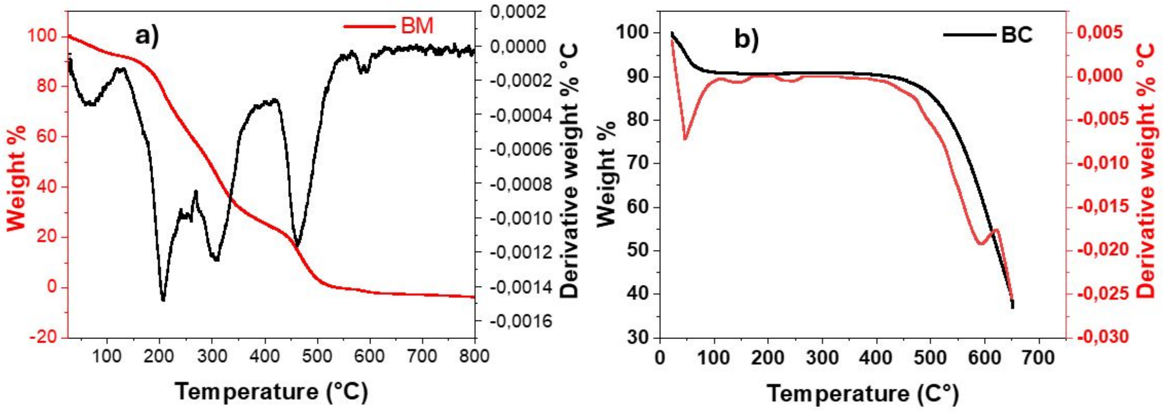

Figure S2. Thermogravimetric analysis (TGA/DTG) of (a) orange peel and bagasse biomass (BM) and (b) $ZnCl_2$-activated biochar. BC: produced at 600 °C for 1 h using $ZnCl_2$ at a 1:3 (w/w) ratio relative to the biomass (BM).

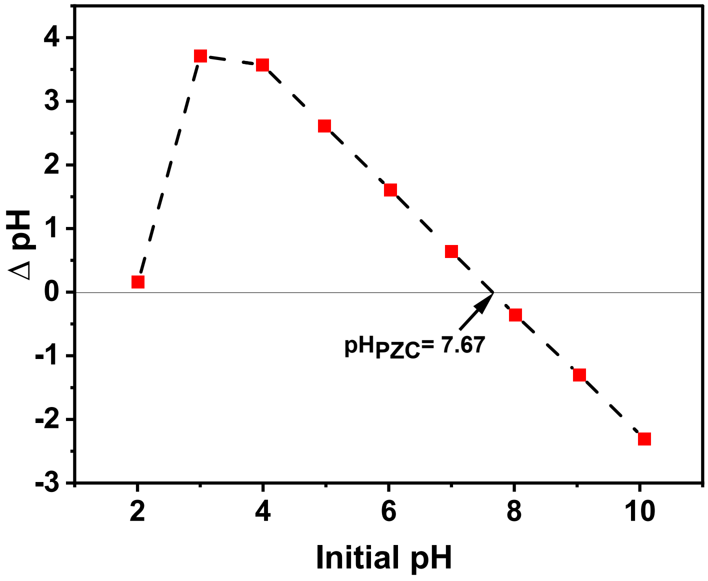

Figure S3. pH at the point of zero charge (pH$_{PZC}$) of $ZnCl_2$-activated biochar (BC). BC: produced at 600 °C for 1 h using $ZnCl_2$ at a 1:3 (w/w) ratio relative to the biomass.

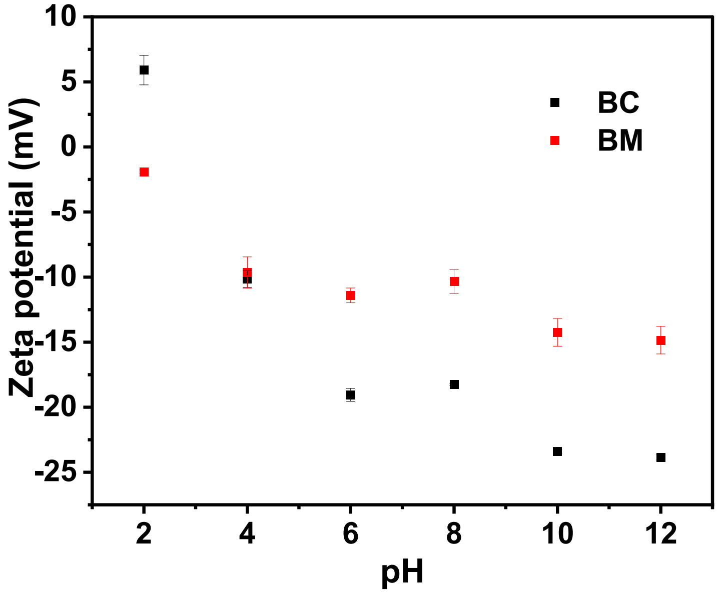

Figure S4. Zeta Potential of $ZnCl_2$-activated biochar (BC) and orange peel and bagasse biomass (BM). BC: produced at 600 °C for 1 h using $ZnCl_2$ at a 1:3 (w/w) ratio relative to the BM.

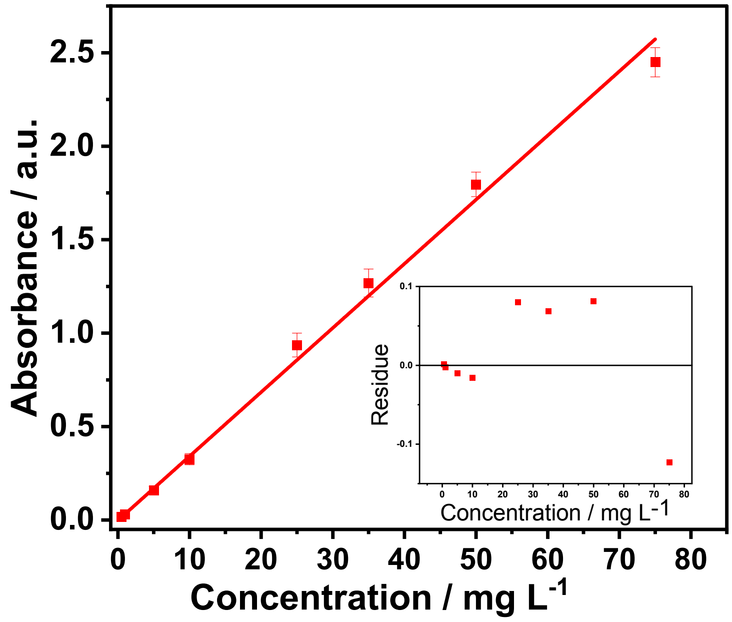

Figure S5. Analytical curve of enrofloxacin over the concentration range of 0.5–75 mg L^{-1}.

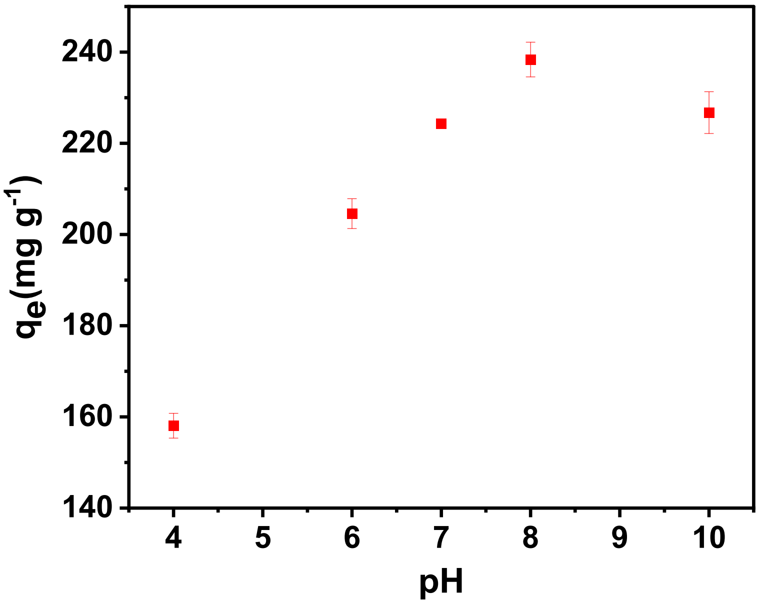

Figure S6. Effect of pH on the adsorption capacity of enrofloxacin (ENR) by $ZnCl_2$-activated biochar. Experimental conditions: initial ENR concentration of 500 mg L^{-1}, adsorbent mass of 15 mg, agitation for 4 h at 25 °C.

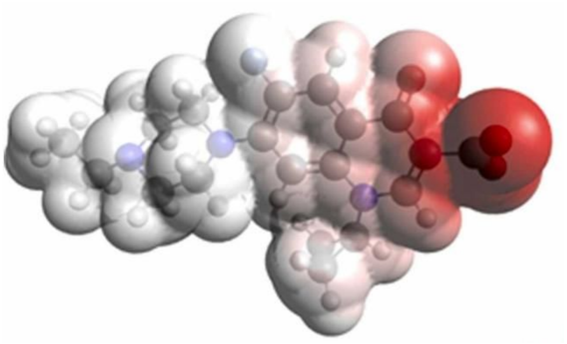

Figure S7. Van der Waals surface colored by electrostatic potential of enrofloxacin. Adapted from Ref. [1] with permission from Elsevier.

Table S1. Textural Properties of Biomass and $ZnCl_2$-Activated Biochar

**Sample**	**Surface Area (m^2 g^{-1})**	**Micropore Volume (cm^3 g^{-1})**	**Average Pore Diameter (nm)**	**Total Pore Volume (cm^3 g^{-1})**
Raw biomass	0.475	0.0000	3.06	0.0006
$ZnCl_2$-activated biochar	1140	0.1589	3.45	1.1862

Reference

[1] dos Santos Silva AA, Bousada GM, Mazzini LFM, et al. Biochar from malt residue: Toward a circular economy for sustainable fluoroquinolone removal in aqueous systems. J Anal Appl Pyrolysis 2024;183. https://doi.org/10.1016/j.jaap.2024.106707.
